# Comparative Genomic Hybridization Selection of Blastocysts for Repeated Implantation Failure Treatment: A Pilot Study

**DOI:** 10.1155/2014/457913

**Published:** 2014-03-23

**Authors:** Ermanno Greco, Sara Bono, Alessandra Ruberti, Anna Maria Lobascio, Pierfrancesco Greco, Anil Biricik, Letizia Spizzichino, Alessia Greco, Jan Tesarik, Maria Giulia Minasi, Francesco Fiorentino

**Affiliations:** ^1^Center for Reproductive Medicine, European Hospital, Via Portuense 700, 00149 Rome, Italy; ^2^GENOMA, Molecular Genetics Laboratory, Via Castel Giubileo 11, 00138 Rome, Italy; ^3^Molecular Assisted Reproduction and Genetics (MAR&Gen) Clinic, Camino de Ronda 2, Bajo, 180 06 Granada, Spain

## Abstract

The aim of this study is to determine if the use of preimplantation genetic screening (PGS) by array comparative genomic hybridization (array CGH) and transfer of a single euploid blastocyst in patients with repeated implantation failure (RIF) can improve clinical results. Three patient groups are compared: 43 couples with RIF for whom embryos were selected by array CGH (group RIF-PGS), 33 couples with the same history for whom array CGH was not performed (group RIF NO PGS), and 45 good prognosis infertile couples with array CGH selected embryos (group NO RIF PGS). A single euploid blastocyst was transferred in groups RIF-PGS and NO RIF PGS. Array CGH was not performed in group RIF NO PGS in which 1-2 blastocysts were transferred. One monoembryonic sac with heartbeat was found in 28 patients of group RIF PGS and 31 patients of group NO RIF PGS showing similar clinical pregnancy and implantation rates (68.3% and 70.5%, resp.). In contrast, an embryonic sac with heartbeat was only detected in 7 (21.2%) patients of group RIF NO PGS. In conclusion, PGS by array CGH with single euploid blastocyst transfer appears to be a successful strategy for patients with multiple failed IVF attempts.

## 1. Introduction

According to ESHRE PGD consortium, repeated implantation failure (RIF) is defined as the absence of a gestational sac on ultrasound at 5 or more weeks after embryo transfer (ET) after 3 embryo transfers with high quality embryos or after the transfer of ≥10 embryos in multiple transfers [1]. Repeated implantation failure can be caused by both maternal and embryonic factors [2]. Intrauterine pathologic conditions, such as polyps, intrauterine adhesions, submucous myomas, and a septated or subseptated uterus, have been demonstrated to disturb embryo implantation [3]. Endometrial receptivity is also decreased with reduced endometrial thickness and/or altered expression of endometrial adhesive molecules [3]. Hydrosalpinx, autoimmune conditions, thrombophilia, inadequate ET methods, or altered life styles are all recognized potential causes of RIF [4]. Sperm DNA damage, zona pellucida hardening, inadequate culture conditions, and suboptimal embryo development can also play a significant role in the etiology of RIF [5]. Development of aneuploid embryos, independently of their morphological quality, is another well-recognized cause of RIF in IVF [6].

Several studies have demonstrated that at least 15% of patients with high-order RIF have an increased frequency of female partner chromosomal abnormalities [7]. Aneuploidy can arise during meiosis or after fertilization. Most meiotic errors are derived from oocytes and the frequency of oocyte aneuploidy increases with advancing female age [8]. Chromosomal abnormalities arising at cleavage stages mostly occur during the first three mitotic divisions, leading to chromosomal mosaicism [9–11]. For these reasons, it has been suggested that the use of preimplantation genetic screening (PGS) to select and transfer chromosomally normal embryos may lead to improved IVF results in this group of patients [12].

Most available data on the impact of PGS on the outcome of IVF in cases of RIF have been obtained with the use of fluorescent* in situ* hybridization (FISH) to assess up to 12 chromosomes in single embryonic blastomeres [13] and are ambiguous and inconclusive. Embryo implantation and live birth rates are not increased after transfer of embryos screened by FISH-based PGS, probably because the limited number of analyzed chromosomes is not sufficient [14, 15]. In fact, it has been demonstrated that aneuploidies may occur in preimplantation embryos in any of the 23 chromosomes indicating that aneuploidy screening of all chromosomes is necessary to determine whether an embryo is chromosomally normal [16–19]. Genome-wide approaches are certainly more comprehensive than FISH (23 compared with ≤12 chromosomes, resp.) and some microarray based methods have shown significantly improved consistency [17–21] and predictive value for aneuploidy diagnosis [22, 23].

The possibility to examine simultaneously all the chromosomes by array comparative genomic hybridization (array CGH) in a few cells or in a single cell [24] provided a new opportunity to evaluate the embryos by PGS in patients with RIF. Array CGH techniques have been recently introduced into current routine PGS laboratory practices. This technique has been adapted for comprehensive molecular cytogenetic analysis of metaphase II oocytes and their polar bodies [25], cleavage stage embryos [17, 26], and blastocysts [27]. In this latter case, biopsy of trophectoderm (TE) cells can have several advantages with respect to biopsy of blastomeres at day 3 of embryo development [28]. Because a larger number of cells can be biopsied from a blastocyst, it is expected that more accurate information can be obtained as compared to one-cell or a few-cell biopsy from cleavage stage embryos, thus reducing the risk of misdiagnosis of embryonic chromosomal mosaicism. Moreover, the TE cell biopsy does not produce any mechanical or functional damage relevant to further development of the biopsied blastocyst [6]. Some scientific studies demonstrate that the cells sampled during TE biopsy are representative of the embryonic inner cell mass (ICM) [27]. Depending on the speed of blastocyst development, the biopsy can be done on day 5, 6, or 7. Only in the first case the transfer of the euploid blastocyst can be performed in the fresh cycle. When the biopsies are carried out on day 6 or 7, it is necessary to cryopreserve the blastocysts by vitrification and delay of the transfer for a subsequent cycle. Initial studies on the clinical use of array CGH in embryos have documented improved identification of abnormalities as well as high pregnancy outcomes following transfer of screened embryos [17, 27, 29, 30]. In particular, a recent study [29], evaluating the efficacy of single embryo transfer (SET) coupled with comprehensive chromosome screening (CCS) in an infertile population, indicated that 23 chromosomes PGS increases ongoing pregnancy rate and reduces the miscarriage rate, compared with traditional blastocyst SET.

In spite of the encouraging data with PGS on cells obtained by TE biopsy, the clinical results of this intervention in a large group of patients with RIF have not yet been clearly demonstrated. Moreover, in the previously published studies more than one euploid blastocyst was transferred and, consequently, many twin gestations were obtained [6, 30]. Additionally one of the most important efforts of IVF practitioners is to reduce the risk of multiple pregnancies maintaining acceptable overall live birth rates following IVF-ET. Elective single embryo transfer is the adopted strategy to reach this scope [31]. Recent studies have demonstrated that single euploid blastocyst transfer gives better clinical results than single morphologically selected blastocyst transfer, both in fresh and frozen-thawed cycles in good prognosis patients [32, 33].

The aim of this study is to assess the clinical pregnancy and implantation rates after transfer of a single euploid blastocyst in a group of patients of <36 years of age and without a history of recurrent miscarriages (RM). The results obtained in this group are compared with a similar group of RIF patients in whom PGS was not performed (negative control) and with a group of good prognosis patients after PGS (positive control).

## 2. Materials and Methods

### 2.1. Inclusion Criteria, Informed Consent, and Ethical Considerations

This study was performed between March, 2012, and March, 2013. A total of 121 couples were involved, including 76 couples with a history of 3–9 (mean 4.9) implantation failures in previous IVF attempts (RIF group) and 45 couples undergoing their first IVF attempt without any detected problem of ovarian reserve and uterine receptivity or sperm quantity and quality (good prognosis group). The couples of the RIF group were further divided into two subgroups: those couples who consented to have their embryos analyzed by array CGH with subsequent transfer of a single euploid blastocyst formed group RIF PGS, whereas group RIF NO PGS (negative control) consisted of couples in whom embryos were not analyzed by array CGH and in whom all available 1-2 blastocysts with the best morphology were transferred. The good prognosis couples who chose array CHG to be performed with their embryos followed by single blastocyst transfer formed group NO RIF PGS (positive control).

All female patients were less than 36 years old. Their ovarian reserve was evaluated before starting ovarian stimulation by determining antral follicle count (AFC), by transvaginal ultrasound on the first days of the cycle (2–5) and by day 3 FSH and anti-Müllerian hormone (AMH) dosage [34]. Patients with abnormal karyotype, uterine abnormalities, autoimmune conditions, thrombophilia, severe endometriosis, and reduced ovarian reserve were excluded from the study.

Seminal fluid examination of male partners was performed after 3–5 days of sexual abstinence according to the World Health Organization (WHO) recommendations [35]. All male patients with severe infertility (<500.000 motile sperm/mL after preparation) or with high sperm DNA fragmentation were excluded [36].

All the three groups were well matched for all relevant male and female clinical parameters (Table 1). A written informed consent was obtained from each couple after counseling about array CGH. The study was approved by the Institutional Review Board of the European Hospital Clinic and GENOMA Laboratory. All experimentations were performed according to the Helsinki Declaration of 1975 and its modifications.

### 2.2. IVF Clinical and Laboratory Protocols

Controlled ovarian stimulation was performed using recombinant FSH (Gonal F, Merck Serono, Geneva, Switzerland) and a long gonadotropin-releasing hormone (GnRH) agonist suppression protocol or GnRH antagonist flexible protocol according to ovarian reserve and AMH values as described elsewhere [37–39]. Recombinant FSH starting dose was calculated taking into account the patient's age, body max index, AFC, and AMH values of the patients. Periodic transvaginal ultrasound scans were performed to assess the number and the mean diameter of the growing follicles. Together with serum estradiol levels, these data were used to adjust the recombinant FSH dose. When at least 3 follicles reached 19 mm in diameter, hCG (Gonasi, 10.000 IU, IBSA, Lodi, Italy) was administered by intramuscular injection. Oocytes were retrieved 36–38 h later by ultrasound-guided transvaginal follicular puncture.

After retrieval oocytes were incubated for 2-3 h at 37°C under the gas phase of 5% O_2_ and 6% CO_2_ before starting the removal of the surrounding cumulus oophorus and corona radiata cells [40, 41]. The oocyte denudation was performed by a brief exposure to 40 IU/mL hyaluronidase solution in fertilization medium (Sage In-Vitro Fertilization, Inc., Trumbull, CT, USA), followed by mechanical removal of all the remaining cumulus and corona cells with the use of plastic pipettes of defined diameters (denuding pipette; COOK Ireland Ltd., Limerick, Ireland). The denudation procedure was completed between 38 and 40 hours after hCG administration, and the oocytes were treated by ICSI immediately thereafter. Particular attention was paid to the removal of all adhering cumulus and coronal cells with the aim to avoid maternal DNA contamination during the amplification steps.

ICSI was performed 38–40 hours after hCG administration, using previously described techniques and instrumentation [42]. Fertilization was considered normal when two clearly distinct pronuclei and two polar bodies were present on day 1, 16–18 h after ICSI as described elsewhere [43]. Embryo culture was carried out in cleavage medium under mineral oil (Sage In-Vitro Fertilization, Inc., Trumbull, CT, USA) up to day 3 of embryo development followed by blastocyst medium (Sage In-Vitro Fertilization, Inc., Trumbull, CT, USA) up to day 5, 6, or 7 at 37°C and under 5% O_2_ and 6% CO_2_. Embryo culture was performed in Embryoscope or in a mini-incubator (SANYO), where all embryos from each patient were kept separately from other couples throughout the culture duration.

### 2.3. Biopsy Procedure and Cells Preparation

On day 3, when the embryos reached the 6–8 cell stage, a noncontact 1.48 u diode laser [27] was used to create a circular 6–9 *μ* diameter opening in the zona pellucida in order to allow the biopsy of 5–10 herniated TE cells on day 5 or 6, depending on the speed of blastocyst development. On the day of biopsy, TE cells were gently aspirated into the biopsy pipette (biopsy pipette; COOK Ireland Ltd., Limerick, Ireland) followed, if necessary, by a laser assisted removal from the body of the blastocyst. The obtained TE cells were washed in sterile phosphate-buffered saline solution (PBS) and then placed in microcentrifuge tubes containing 2 *μ*L of PBS, spinned down for few seconds and sent to GENOMA Laboratory for analysis.

### 2.4. Array CGH Protocol

TE cells were lysed, and genomic DNA was amplified using the SurePlex DNA Amplification System (BlueGnome, Cambridge, UK), according to the manufacturer's instructions. Whole Genome Amplification (WGA) products were processed as reported elsewhere [17] according to the BlueGnome 24sure V3 protocol (available at http://www.cytochip.com/). Briefly, WGA products were fluorescently labelled and competitively hybridized to 24sure V3 arrays (BlueGnome, Cambridge, UK) with a matched control in an array CGH experiment format. A laser scanner InnoScanw 710 AL (INNOPSYS, Carbonne, France) was used to excite the hybridized fluorophores and read and store the resulting images of the hybridization. Scanned images were then analysed and quantified by algorithm fixed settings in BlueFuse Multi Software (BlueGnome, Cambridge, UK), a software package that performed the steps of grid placement, quantification, normalization, and postprocessing automatically. The whole procedure was completed within 12–24 h, and the results were obtained in time for an embryo transfer at day 6, in a fresh cycle.

### 2.5. Embryo and Blastocyst Grading

Embryo morphology was checked on days 2 and 3 using the scoring system reported elsewhere [40]. Briefly, for each embryo, the number and size of the blastomeres were observed, as well as the percentage of anucleated fragments. Cleaved embryos with no more than 20% of their volume occupied by fragments and with equal-sized blastomeres were considered type A. When the percentage of the volume filled with fragments was between 20% and 50%, the embryos were considered type B. Finally, when >50% fragments were present, embryos were considered type C.

Embryos reaching the blastocyst stage were graded by using the system of Gardner and Schoolcraft [44]. Blastocysts were given a number based on the degree of expansion and hatching status (from 1 to 6): (1) early blastocyst: the blastocoel accounts for less than one-half of the volume of the embryo; (2) blastocyst: the blastocoel occupies more than one-half of the volume of the embryo; (3) full blastocyst: the blastocoel fills the embryo completely; (4) expanded blastocyst: the blastocoel is now larger than the early embryo, and the zona pellucida has begun to thin; (5) hatching blastocyst: TE cells have begun to herniate through the zona pellucida; and (6) hatched blastocyst: the blastocyst has completely escaped from the zona pellucida.

For fully developed blastocysts (grades 3–6), a second scoring step was performed under an inverted microscope to assess the inner cell mass and the trophectoderm. For the ICM, the following descriptions are used: (a) tightly packed with many cells, (b) loosely grouped with several cells, and (c) very few cells. For the TE, the following grading is used: (a) many cells forming a cohesive epithelium, (b) few cells forming a loose epithelium, and (c) very few large cells.

### 2.6. Blastocyst Vitrification and Warming

Vitrification procedure was used to cryopreserve all surplus blastocysts, both euploid ones, for later use, and aneuploid ones whose storage is dictated by the Italian law which forbids any embryo destruction. All embryos that reached the blastocyst stage on day 6 or 7 were also vitrified. Vitrification was carried out with the use of the Kuwayama protocol with Cryotop as support as previously described [45]. In brief, blastocysts were placed in equilibration solution (Kitazato Vitrification Kit, BioPharma, Shizuoka, Japan) containing 7.5% ethylene glycol and 7.5% dimethyl sulfoxide for 15 minutes at room temperature and then moved to a vitrification solution composed of 15% ethylene glycol, 15% dimethyl sulfoxide, and 0.5 mol/L sucrose for 30 to 60 seconds. Individual blastocysts were loaded onto the polypropylene strip of the Cryotop in a volume of <0.1 *μ*L, quickly plunged into liquid nitrogen, capped with a protective cover, and stored in a liquid N_2_ storage tank at −196°C.

Warming was carried out with the use of the Kuwayama protocol as previously described [45]. In brief, warming was performed by placing the Cryotop in a thawing solution (Kitazato Warming Kit, BioPharma, Shizuoka, Japan) of 1 mol/L sucrose for 45 to 60 seconds at 37°C. Blastocysts then were transferred to a dilution solution of 0.5 mol/L sucrose for 3 minutes, followed by washing with medium without sucrose for 5 minutes. The surviving blastocysts were incubated for 4 hours before their transfer to the uterus. Endometrial preparation was carried out as described previously [46].

### 2.7. Blastocyst Transfer, Luteal Phase Support, and Pregnancy Determination

Single frozen-thawed embryo transfer (FET) was performed in patients prepared by combining gonadotropin-releasing hormone agonist and estrogen pills (Progynova, Bayer, New Zealand Limited, Auckland) or in a spontaneous cycle. Fresh single embryo transfer (SET) was performed in the morning of day 6 after ICSI. All transfer procedures were carried out with the use of a catheter (Wallace, Smits-Medical, Dublin, Ireland) under direct ultrasound guidance as previously described [47]. In groups RIF PGS and NO RIF PGS only euploid blastocysts were selected for transfer. If several euploid blastocysts were available in these two groups, the best-morphology one was selected for transfer. In group RIF NO PGS (without ploidy evaluation), 1-2 best-morphology blastocysts were transferred. No blastocyst transfer was done with endometrium thickness of <7 mm.

Intramuscular administration of progesterone in oil (Prontogest, IBSA, Lodi, Italy) was initiated 6-7 days before embryo transfer and continued until the first serum *β*-hCG determination. Biochemical pregnancy was confirmed by the detection of increasing *β*-hCG levels 9 days after blastocyst transfer. Clinical pregnancies were defined as those showing the presence of an intrauterine gestational sac determined by transvaginal ultrasound examination at 7 weeks of gestation. Implantation rate was defined as the number of gestational sacs per transferred embryos [48].

### 2.8. Statistical Methods

Required sample size was estimated to evaluate the implantation rate after the transfer of a single euploid blastocyst in patients with only repeated implantation failure without advanced maternal age or previous miscarriages. Considering a prevalence of 50% of implantation rate, with an error of 15% and 95% confidence interval, 43 couples are needed. The same number of good prognosis couples is considered as positive control group (C). Only 33 couples were available for the negative control group (B), but the number of embryos transferred in this group was similar to that in groups A and C.

In order to compare the maternal age, FSH, AMH, number of antral follicles, sperm count, sperm motility, and sperm morphology between the three groups ANOVA was used. Chi-square test, or Fisher's exact test when necessary, was used to compare categorical data. Bonferroni correction was used in post hoc analysis comparing only groups A versus B and groups A versus C. Exact confidence interval at 95% (95% CI) was reported regarding the principal outcome. A *P* value <0.05 was considered statistically significant. Stata 12.1 was used for all analysis.

## 3. Results

All clinical parameters, including age, day 3 serum FSH concentration, serum AMH concentration and days 2–5 AFC for the female partners, and sperm count, motility, and morphology for the male partner, were similar in groups A (*n* = 43), B (*n* = 33), and C (*n* = 45) (Table 1).

In group RIF PGS, 645 oocytes were collected; 530 of them were in metaphase II stage (82.2%) and all of them were injected and 433 fertilized normally (81.7%) resulting in 392 embryos (90.5). In group RIF NO PGS, 519 oocytes were collected; 454 of them were metaphase II (87.5%) and all of them were injected and 373 fertilized normally (82.2%) resulting in 337 embryos (90.3%). In group NO RIF PGS, 681 oocytes were collected; 556 of them were metaphase II (81.6%) and all of them were injected and 451 fertilized normally (81.1%) resulting in 408 embryos (90.5%) (Table 2).

In group RIF PGS, day 3 embryo quality was A = 236 (60.2%), B = 105 (26.8%), and C = 51 (13.0%). A total of 190 blastocysts were obtained (48.5%): 83 blastocysts were obtained on day 5 (43.7%), 96 on day 6 (50.5%), and 11 on day 7 (5.8%). In group RIF NO PGS day 3 embryo quality was A = 210 (62.3%), B = 64 (19.0%), and C = 63 (18.7%). A total of 171 blastocysts were obtained (50.7%): 87 blastocysts were obtained on day 5 (50.9%), 51 on day 6 (29.8%), and 33 on day 7 (19.3%). In group NO RIF PGS, day 3 embryo quality was A = 246 (60.3%), B = 112 (27.5%), and C = 50 (12.3%). A total of 257 blastocysts were obtained (63.0%): 108 blastocysts were obtained on day 5 (42.0%), 125 on day 6 (48.6%), and 24 on day 7 (9.3%) (Table 2).

In groups RIF PGS and NO RIF PGS, a total of 447 blastocysts were obtained on days 5–7 (Table 2). All blastocysts were biopsied in both of these groups. The percentage of embryos reaching the blastocyst stage in group RIF PGS was lower as compared to group NO RIF PGS, and no statistical difference was observed in the other above-mentioned biological parameters between the two groups (*P* > 0.05) (Table 2). In group RIF NO PGS the total number of blastocysts (days 5–7) was 171. Similar to group RIF PGS, the percentage of blastocysts in group RIF NO PGS was lower than that in group NO RIF PGS (Table 2). No blastocyst was biopsied in this group.

In group RIF PGS, array CGH yielded interpretable results for 182 out of the 190 blastocysts, leading to a diagnostic efficiency of 95.8%. In group NO RIF PGS, array CGH yielded interpretable results for 245 out of the 257 blastocysts, with a diagnostic efficiency of 95.3% (Table 3).

In group RIF PGS, 84 blastocysts were classified as euploid (46.2%), whereas 98 were classified as aneuploid, resulting in an abnormality rate of 53.8%. Of the aneuploid blastocysts, 35.7% (*N* = 35) were complex aneuploidies, 22.4% (*N* = 22) carried two chromosome errors, 19.4% (*N* = 19) trisomies, 21.4% (*N* = 21) monosomies, and 1.0% (*N* = 1) mosaicisms (Table 3). In group NO RIF PGS, 127 blastocysts were classified as euploid (51.8%), whereas 118 were classified as aneuploid, resulting in an abnormality rate of 48.2%. Of the aneuploid blastocysts, 27.1% (*N* = 32) were complex aneuploidies, 25.4% (*N* = 30) carried two chromosome errors, 21.2% (*N* = 25) trisomies, 22.9% (*N* = 27) monosomies, and 3.4% (*N* = 4) mosaicisms. No statistical differences were observed in aneuploidy rates and types of aneuploidies between the two groups (*P* = 0.245; *P* = 0.173) (Table 3).

Forty-one couples received a single euploid blastocyst replacement in group RIF PGS and 44 in group NO RIF PGS. In group RIF PGS, 15 patients received a single fresh blastocyst transfer (36.6%) and 26 patients received a single frozen-thawed blastocyst transfer (63.4%), 14 in natural cycle and 12 after endometrial preparation with exogenous estrogen (Table 4). In group NO RIF PGS, 19 patients received a single fresh blastocyst transfer (43.2%) and 25 patients received a single frozen-thawed blastocyst transfer (56.8%), 12 in natural cycle and 13 after endometrial preparation (Table 4). All the cryopreserved blastocysts survived after thawing in both groups (100%).

In group RIF NO PGS, 41 blastocysts were transferred and all of them in the fresh state. There were 25 single embryo transfers and 8 double embryo transfers (Table 4).

*β*-hCG test was positive for 34 couples in group RIF PGS (82.9%) and for 37 in group NO RIF PGS (84.1%) (*P* = 0.331). In contrast, only 9 couples (27.3%) had a positive *β*-hCG test in group RIF NO PGS, significantly less as compared with both group RIF PGS and group NO RIF PGS (*P* < 0.001) (Table 4).

**Figure 1:**
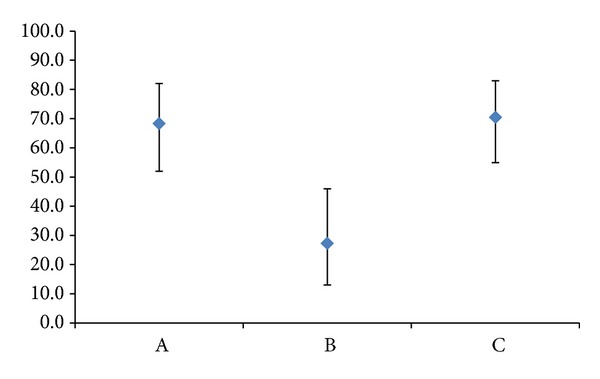
Implantation rates in groups RIF PGS (A), RIF NO PGS (B), and NO RIF PGS (C).

Ultrasound examination, which took place 7 weeks after ET, revealed one monoembryonic sac with heartbeat in 28 patients of group RIF PGS, in 7 patients of group RIF NO PGS, and in 31 patients of group NO RIF PGS. Clinical pregnancy and implantation rates, respectively, were 68.3% and 68.3% in group RIF PGS, 22.0% and 21.2% in group RIF NO PGS, and 70.5% and 70.5% in group NO RIF PGS (Figure 1). In group RIF PGS, 2 pregnancies were biochemical, 3 anembryonic, and one tubal. In group RIF NO PGS, 2 pregnancies were biochemical, none anembryonic, and none tubal. In group NO RIF PGS, 4 pregnancies were biochemical, 2 anembryonic, and none tubal. There were no spontaneous abortions in any group (Table 4).

There were no statistically significant differences in the clinical pregnancy and implantation rates between fresh and frozen cycles for any group (Table 4).

In contrast only 7 clinical pregnancies were achieved in group RIF NO PGS, all of them monoembryonic. Clinical pregnancy rate (21.2%) and implantation rate (22.0%) in group RIF NO PGS were significantly lower (*P* < 0.001) as compared to both group RIF PGS and group NO RIF PGS.

All patients with clinical pregnancy in any group have delivered a healthy child.

## 4. Discussion

Repeated implantation failure (RIF) refers to a situation when good quality embryos fail to implant in several subsequent IVF treatment cycles. The mechanism of embryo implantation failure is poorly understood, but it is clear that it can involve both maternal and embryonic factors.

Failure of implantation due to embryonic causes is associated with either genetic abnormalities or other factors intrinsic to the embryo that impair its ability to develop in the uterus, to hatch, and to implant. A high incidence of complex chromosome abnormalities has been discovered in cleaving embryos from patients with RIF [49, 50].

This is the first study to show that embryo selection by array CGH, performed at the blastocyst stage, has the potential to improve the implantation rate and the success rate of IVF cycles in patients with RIF. In fact, the group of couples with single euploid blastocyst transfer compares favourably with the clinically comparable group of couples in whom the transferred blastocysts were selected merely on the base of morphology (negative control), even though more blastocysts were transferred in the latter group. These results are not unexpected since it has been shown that embryo development to blastocyst stage does not represent by itself an absolute selective barrier against chromosome errors [51] even though the rate of aneuploidy is significantly lower for blastocysts than for embryos at earlier stages (38.8% versus 51%) [52].

Moreover, the success rate of IVF after single euploid blastocyst transfer gives similar results for couples with RIF and for good prognosis couples in their first IVF attempt (positive control). This latter comparison demonstrates, for the first time, that embryo aneuploidies are by far the main cause of RIF as compared with other possible etiologies. The present data also confirm that trophectoderm biopsy does not impair the implantation potential, in agreement with a previous study [53]. The identification of the most viable embryos within a cohort is one of the main goals in IVF in order to perform a single embryo transfer and avoid multiple pregnancies. A recent study reports that the cohort size is not significantly associated with the aneuploidy rate [54]. Several morphological scoring systems have been designed to select the most viable embryos in a cohort, by analyzing pronuclear-stage zygotes [43], cleavage-stage embryos [42], and blastocysts [44]. Our study, in agreement with others, shows that morphological criteria of embryo selection are not fully representative of the genetic health of the embryo at the blastocyst stage [55, 56]. For this reason, it appears to be difficult to further improve clinical pregnancy rates with purely morphological criteria, even though blastomere fragmentation and multinucleation in early cleaving embryos have been shown to be associated with an increased risk of anomalies in chromosome segregation, leading to chromosomal aberrations [57, 58]. This is even more evident in poor prognosis patients such as those with advanced maternal age (AMA), with recurrent abortion (RA), and, as in the present study, with RIF.

In this study, all the embryos were cultured until the blastocyst stage. The number of blastocysts available on days 5, 6, and 7 was similar for groups RIF PGS and NO RIF PGS. On the other hand, less blastocysts developed to day 6 and more blastocysts developed to day 7 in group RIF NO PGS. These differences may be due to the fact that embryos of group NO RIF PGS were not biopsied. However, the mechanism of these differences is not clear for the time being. It has been demonstrated that only embryos with highest implantation potential are able to achieve this stage and that embryos affected by chaotic mosaicism have a reduced capacity for forming a blastocyst [59, 60]. This can explain the reduced blastocysts development in RIF patients with respect to good prognosis couples obtained in the present study. On the other hand, many studies have confirmed that chromosome abnormalities and mosaicism remain common at the blastocyst stage, although aneuploidy rates are reduced compared with the cleavage stages [11]. There has been an increasing interest in defining the types of chromosome errors compatible with blastocyst development. The results of this study confirm that even embryos with the most severe chromosomal abnormalities are often capable of developing up to the blastocyst stage [10], although the aneuploidy rate in blastocysts might be lower than that obtained, using similar methods, in early cleaving embryos [61]. These observations confirm and extend previously published data on blastocyst biopsy and array CGH testing [30, 62].

The accuracy and efficiency of the identification of blastocyst chromosome abnormalities by TE cell biopsy have been validated by different studies showing that TE samples provide accurate information of the chromosome constitution of the inner cell mass in the vast majority of cases [63]. The risk of misdiagnosis of chromosomal mosaicism is reduced by the analysis of several cells obtained by TE biopsy (5–10), and it has been demonstrated that most mosaic blastocysts do not contain any normal cells [62]. Clinical data suggest that an approach combining blastocyst biopsy and comprehensive chromosome screening using array CGH or microarray CGH may represent the optimal approach for preimplantation genetic screening [15, 52].

In the investigation of chromosome abnormalities at the blastocyst stage, the presence of aneuploidy for three or more chromosomes has been defined as complex chromosome abnormality [6]. Since complex chromosome abnormalities have a relatively low incidence in oocytes, their presence in cleaving embryos and blastocysts is likely to be of postzygotic origin, resulting from abnormal mitotic divisions of embryonic cells [6]. The factors responsible for these abnormalities are not known, and it has been suggested that the lack or dysfunction of cell cycle checkpoints at different cleavage stages of embryo development may be implicated [11]. Complex chromosome abnormalities can be identified more accurately with the use of array CGH as compared with FISH because array CGH is capable of providing data about the whole genome, whereas the capacity of FISH is relatively limited. There appears to be no predilection of any individual chromosome to be involved in a complex chromosome abnormality. Consequently, the limitation of evaluating one, two, or a few chromosomes, as is the case in most FISH protocols, bears the risk of missing an important complex chromosome abnormality or of misinterpreting it as a simple aneuploidy. This may explain the contradictory results obtained by FISH in RIF patients [6].

Our study was intended to be a preliminary and rapid clinical evaluation for a new treatment option in RIF patients with a high number of previous failed attempts in order to obtain successful clinical results avoiding the potential risk of multiple ovarian stimulations. To the best of our knowledge, this is also the largest genetic study on embryos from RIF patients without advanced maternal age or multiple abortions.

Unlike previous studies [64, 65], our data do not highlight any detrimental effect of day 6 transfer on the blastocyst implantation rate. Larger and RCT studies are needed to confirm these preliminary observations.

In conclusion, the results of this study show that array CGH has the potential to provide high rates of embryo implantation after transfer of a single euploid blastocyst in patients with a history of RIF following transfer of good-morphology embryos.

Thus, the combined use of array CGH and single blastocyst transfer can provide an efficient tool for improving IVF clinical outcomes in RIF patients without increasing the number of transferred embryos and the risk of unwished multiple pregnancies. In a wider perspective, this technique can also be used in patients who, independently of a RIF history, wish to limit the number of transferred embryos to a single one for different personal, social, or economic reasons.

## Figures and Tables

**Table 1:** Clinical parameters analyzed for the three groups (SD, standard deviation).

	Group RIF PGS (*n* = 43)	Group RIF NO PGS (*n* = 33)	Group NO RIF PGS (*n* = 45)	*P*
Mean female age ± SD	32.8 ± 3.1	31.5 ± 2.9	31.7 ± 2.9	NS
Mean FSH ± SD (day 3, nv 3–10 mUI/mL)	7.8 ± 1.7	7.7 ± 0.7	8.1 ± 1.8	NS
Mean AMH ± SD (nv 0, 2–5, 5 ng/mL)	4.1 ± 1.1	5.2 ± 2.4	4.6 ± 1.2	NS
Mean number of antral follicles ± SD	12.4 ± 1.9	12.9 ± 2.8	13.8 ± 2.1	NS
Mean sperm count (M/mL) ± SD	9.8 ± 2.1	14.4 ± 9.2	10.1 ± 2.0	NS
Mean sperm motility (%) ± SD	50.7 ± 16.8	47.4 ± 15.5	51.2 ± 16.9	NS
Mean sperm morphology (%) ± SD	5.5 ± 2.3	6.7 ± 2.9	5.1 ± 2.1	NS

**Table 2:** Oocytes, embryos, and blastocysts obtained in the three groups.

	Group RIF PGS (*n* = 43)	Group RIF NO PGS (*n* = 33)	Group NO RIF PGS (*n* = 45)	*P*
Number of oocytes	645	519	681	
Metaphase II	530 (82.2%)	454 (87.5%)	556 (81.6%)	0.014 A versus B 0.026* A versus C 1.00*
Fertilized oocytes	433 (81.7%)	373 (82.2%)	451 (81.1%)	0.912
Embryos	392 (90.5%)	337 (90.3%)	408 (90.5%)	0.996
Day 3 grade A	236 (60.2%)	210 (62.3%)	246 (60.3%)	0.013
Day 3 grade B	105 (26.8%)	64 (19.0%)	112 (27.5%)	A versus B 0.026*
Day 3 grade C	51 (13.0%)	63 (18.7%)	50 (12.3%)	A versus C 1.00*
Blastocysts	190 (48.5%)	171 (50.7%)	257 (63.0%)	<0.001 A versus B 1.00* A versus C 0.001*
Day 5	83 (43.7%)	87 (50.9%)	108 (42.0%)	<0.001
Day 6	96 (50.5%)	51 (29.8%)	125 (48.6%)	A versus B 0.001*
Day 7	11 (5.8%)	33 (19.3%)	24 (9.3%)	A versus C 0.770*

*After Bonferroni's correction.

**Table 3:** Array comparative genomic hybridization results.

	Group RIF PGS (*n* = 43)	Group RIF NO PGS (*n* = 33)	Group NO RIF PGS (*n* = 45)	*P*
Blastocysts with amplification (efficiency %)	182/190 (95.8%)	Not applicable	245/257 (95.3%)	0.817
Euploidy	84/182 (46.2%)	Not determined	127/245 (51.8%)	0.245
Aneuploidy	98/182 (53.8%)	Not determined	118/245 (48.2%)	
Complex aneuploidy	35 (35.7%)	Not determined	32 (27.1%)	0.610
Two chromosome errors	22 (22.4%)	Not determined	30 (25.4%)	
Trisomic	19 (19.4%)	Not determined	25 (21.2%)	
Monosomic	21 (21.4%)	Not determined	27 (22.9%)	
Mosaicism	1 (1.0%)	Not determined	4 (3.4%)	

**Table 4:** Clinical results.

	Group RIF PGS (*n* = 43)	Group RIF NO PGS (*n* = 33)	Group NO RIF PGS (*n* = 45)	*P*
Total single embryo transfer Total double embryo transfer	41 0	25 8	44 0	
Total fresh embryo transfer	15 (36.6%)	33 (100%)	19 (43.2%)	<0.001 A versus B 0.001* A versus C 1.00*
Total frozen embryo transfer	26 (63.4%)	0	25 (56.8%)	<0.001 A versus B 0.001* A versus C 1.00*
Frozen embryo transfer in natural cycle	14 (53.8%)		12 (48.0%)	0.676
Frozen embryo transfer with endometrial preparation	12 (46.2%)		13 (52.0%)	
Total embryos transferred bHCG positive	41 34 (82.9%)	41 9 (27.3%)	44 37 (84.1%)	<0.001 A versus B 0.001* A versus C 1.00*
Implantation (IR)	28 (68.3%)	9 (22.0)	31 (70.5%)	<0.001 A versus B 0.001* A versus C 1.00*
Clinical pregnancy (CPR)	28 (68.3%)	7 (21.2%)	31 (70.5%)	0.609
Biochemical pregnancy	2 (4.9%)	2 (6.1%)	4 (9.1%)	
Anembryonic pregnancy	3 (7.3%)	0	2 (4.5%)	
Tubal pregnancy Spontaneous abortion	1 (2.4%) 0	0 0	0 0	

*After Bonferroni's correction.
